# High initial target blood concentration in target‐controlled infusion: A randomized controlled trial

**DOI:** 10.1002/cre2.632

**Published:** 2022-07-22

**Authors:** Toshiaki Fujisawa, Shigeru Takuma, Yukie Nitta

**Affiliations:** ^1^ Department of Dental Anesthesiology, Faculty of Dental Medicine and Graduate School of Dental Medicine Hokkaido University Sapporo Japan

**Keywords:** conscious sedation, infusion pump, pharmacokinetics, propofol

## Abstract

**Objective:**

Our previously modified propofol intravenous sedation (IVS) method using a target‐controlled infusion (TCI) pump with initial target blood concentration (TBC) set at 2.2 μg/ml enables the prediction of the personal optimal intraoperative TBC during induction with a minimal gap. This study aimed to verify whether this method can be useful in case of higher initial TBCs to reduce induction time.

**Methods:**

Forty‐five patients scheduled to undergo oral surgery under IVS with propofol were randomly divided into three groups (group 1, TCI started with TBC set at 2.2 μg/ml; group 2, TBC was set at 2.6 μg/ml; group 3, TBC was set at 3.0 μg/ml). Immediately after reading the calculated brain concentration when the target sedation was achieved (value A), the initial TBC was manually reset to value A. We manually controlled the intraoperative TBC to maintain moderate sedation, according to the clinical signs and bispectral index values. Of the regulated TBC values, the value farthest from value A was defined as value B. The maximum discrepancy between values B and A and the induction time were compared among the three groups.

**Results:**

The maximum discrepancy (mean ± standard deviation [SD]) was significantly larger in group 3 (1.0 ± 1.3 μg/ml, *p* = .005) and group 2 (0.8 ± 0.2 μg/ml *p* = .008) than in group 1 (0.5 ± 0.3 μg/ml). The induction time (mean ± SD) was significantly shorter in group 3 (124 ± 126 min, *p* = .004) and group 2 (135 ± 33 min, *p* = .006) than in group 1 (245 ± 1913 min). With the initial TBC set at 2.6 μg/ml, the maximum discrepancy was large at 0.8 μg/ml, but with a small SD (0.2 μg/ml).

**Conclusion:**

Considering this discrepancy, this method with an initial TBC set at 2.6 μg/ml may be acceptable for clinical use for moderate sedation (UMIN 000017197).

## 1 INTRODUCTION

Due to variation in the sensitivity to sedatives, it is difficult to predict an individual's response to sedatives (Committee on Quality Management and Departmental Administration, 2020) or the optimal dosage of a sedative during intravenous sedation (IVS). Therefore, it will be clinically helpful if the optimal intraoperative dosage for each individual can be predicted during induction.

Recently, propofol infusion by a target‐controlled infusion (TCI) system for conscious sedation in dental patients has been widely used (Leitch et al., 2003; Oei‐Lim et al., 1997, 1998). A TCI system controls the blood concentration of drugs to a targeted value with a computer‐controlled infusion pump that uses pharmacokinetic stimulation models in its calculation to theoretically achieve and maintain a stable drug concentration (Oei‐Lim et al., 1998). Currently, TCI systems are approved for general anesthesia use and/or IVS in at least 96 countries (Absalom et al., 2016).

In previous studies on dental patients and volunteers, we investigated the usefulness of a modified propofol IVS method using a TCI pump (Fujisawa et al., 2004). We started the TCI after the initial target blood concentration (TBC) had been set at 2.2 μg/ml. Immediately after the TBC for moderate sedation was achieved, the TBC was manually reset to the same value (value X) as the calculated brain (effect‐site) concentration displayed at that time. Thereafter, the intraoperative TBC was manually regulated according to the clinical signs to maintain the desired sedation. Although there was a wide range of X values due to individual sensitivity, this method was capable of maintaining the sedation level with small adjustments in TBC from value X during the operation. This method enabled the prediction of the optimal intraoperative propofol TBC for each individual with minimal discrepancy during induction. This was useful because the calculated blood concentration could be easily and reliably maintained by a TCI pump (Fujisawa et al., 2004; Takuma et al., 2005).

IVS is useful for the reduction of psychological stress that could lead to perioperative vago‐vagal reflex, especially in patients with dental phobia. However, it does not reduce the anxiety before the onset of sedation. We routinely use this method in a daily clinical setting and sometimes attempt to start TCI with an initial TBC of over 2.2 μg/ml for patients with dental fear to reduce the induction time. Our working hypothesis is that even if this method was used with a higher initial TBC, it would still enable the prediction of optimal intraoperative propofol TBCs with minimal discrepancy and maintain cardiovascular and respiratory stability during IVS, as well as enable a shorter induction time.

## 2 METHODS

The present study was a single‐blind, parallel‐group randomized trial. It was approved by the local ethics committee (Institutional Review Board of Hokkaido University Hospital, Clinical Study Code: 012‐0298) and registered in the University Hospital Medical Information Network (UMIN) in Japan (registration number: 000017197). This study was conducted in accordance with the guidelines of the Helsinki Declaration and adheres to the applicable statement of consolidated standards of reporting trials.

A total of 45 patients aged 18–55 years, who were scheduled to undergo oral surgery under a combination of IVS and local anesthesia administered by the Department of Dental Anesthesiology in our outpatient setting in Hokkaido University Hospital between April 2010 and January 2011, were included in this study.

Patients taking sedatives as regular medication and those with hepatic or renal dysfunction, neuromuscular disorders, Broca Index < −30% or >30%, or who were advised to use narcotics during surgery were excluded. The participants were divided into three groups: group 1—the TCI was started with the initial TBC set at 2.2 μg/ml; group 2—the initial TBC was set at 2.6 μg/ml; and group 3—the initial TBC was set at 3.0 μg/ml.

The random allocation was done by a researcher not involved in the measurement and analysis of variables or management of IVS. An investigator who was responsible for the measurement of the variables were informed of the allocation on the day of the operation to ensure concealment of the random allocation. Written informed consent was obtained from all patients.

### 2.1 TCI pump for propofol and infusion pattern used in this study

**Figure 1:**
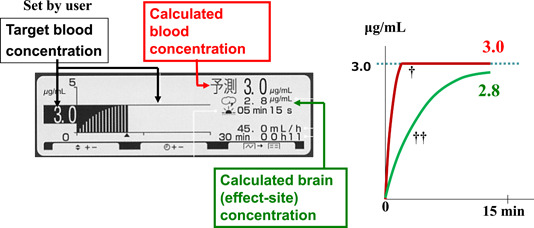
Display panel of the TCI pump for propofol and outline of changes in the calculated blood and brain (effect‐site) concentrations during induction. When TCI is started with an initial TBC set at a certain targeted value (e.g., 3.0 μg/ml), the calculated blood concentration reaches the same value as the targeted value in about 15 s and the calculated brain (effect‐site) concentration reaches the same value as the targeted value in 15 min. ^†^Curve of calculated blood concentration; ^††^Curve of calculated brain (effect‐site) concentration. TBC, target blood concentration; TCI, target‐controlled infusion.

The TCI pump TE‐371™ (Terumo Co.) was used as the TCI pump for propofol in this study. Diprifusor™ is a TCI system with a multicompartment pharmacokinetic model, known as the Marsh model (Marsh et al., 1991), which is preloaded in the pump. The Marsh model is a common pharmacokinetic model used in TCI systems to describe propofol concentration dynamics in the blood. When TCI was started with the initial TBC set at a certain targeted value (e.g., 3.0 µg/ml), a loading dose of propofol bolus was infused followed by continuous intravenous infusion. The infusion rate was then automatically regulated to allow the calculated blood concentration to maintain at 3.0 µg/ml. The pump display panel and outline of changes in the calculated blood and brain (effect‐site) concentrations during induction are shown in Figure 1.

The TCI pump TE‐371™ model has been discontinued, and this TCI system was replaced by a more recent model, the Terufusion syringe pump SS type 3TCI™. The method of operation is basically the same as that of the previous model.

### 2.2 Study design

**Figure 2:**
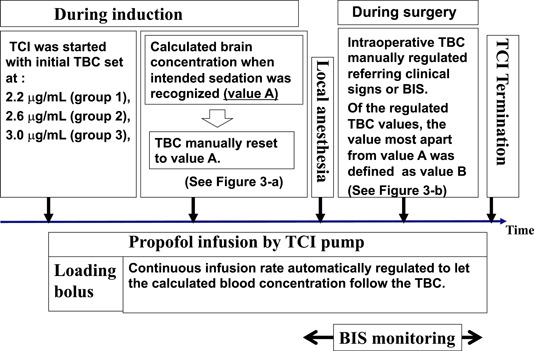
Study protocol. BIS, bispectral index; TBC, target blood concentration; TCI, target‐controlled infusion.

Figure 2 shows the study protocol. The intended sedation level was a state where the patients' eyes were closed, but the patients still responded to mild verbal commands (moderate sedation level). The TCI was started with the initial TBC set at each of the three concentrations described above.

**Figure 3:**
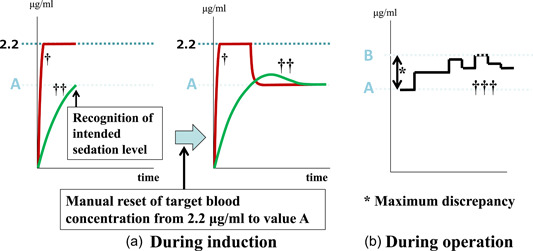
Method to calculate the “maximum discrepancy” (|value B − value A|) in case of initial target blood concentration (TBC) set at, for example, 2.2 μg/ml. ^†^Curve of calculated blood concentration; ^††^Curve of calculated brain concentration; ^†††^TBC manually regulated.

When the initial TBC had been set at, for example, 2.2 μg/ml, the loading dose of bolus infusion (0.5 mg/kg of propofol) was administered. The infusion rate was then automatically regulated to allow the calculated blood concentration to maintain at 2.2 μg/ml. Immediately after the moderate sedation level was achieved during induction, the TBC was manually reset to the same value as the calculated brain (effect site) concentration displayed at that time (conveniently called value A) (Figure 3a). Thereafter, to maintain moderate sedation, the intraoperative TBC was manually regulated by monitoring a clinical sign involving the closure of the eyes, albeit with a rapid response to the name spoken in a normal tone; simultaneously, the bispectral index (BIS) values were also maintained between 75 and 85 to achieve moderate sedation. Of the regulated intraoperative TBC values, the value that was farthest from value A was defined as value B (see Figure 3b). The absolute value of the difference between value B and value A (|value B − value A|) was defined as the “maximum discrepancy.” Initial TBC values were blinded to all patients.

### 2.3 Primary and secondary endpoints

The primary endpoint of this study was the “maximum discrepancy.” The group with a maximum discrepancy that was not significantly different from that of group 1 was considered as the group in which the TCI system was capable of predicting the personal optimal intraoperative TBC during induction with a minimal discrepancy range. The secondary endpoints were as follows: (1) the calculated brain concentration when moderate sedation was achieved during induction (value A), (2) time required from TCI initiation to moderate sedation (induction time), (3) maximum rate of decrease (baseline value − lowest value/baseline value) in the blood pressure, heart rate, and SpO_2_.

### 2.4 Power analysis and statistics

In this study, we regarded an increase of 0.26 μg/ml or greater over the intergroup difference in maximum discrepancy as a significant change. A calculation of the required sample size with *α* = .05 and *β* = .2 showed that 15 members were needed in each group. Therefore, the number of patients in the present study was set at 45.

The measured parameters were compared among the three groups. The JMP® software (SAS Institute, Inc.) was used for the statistical analysis. An analysis of variance with a post hoc test (Dunnett) was used to assess the differences in the patients' characteristics and compare the various evaluation parameters as continuous variables. The *χ* ^2^ test was used for the comparison of categorical data. The level of significance was set at *p* < .05.

## 3 RESULTS

**Figure 4:**
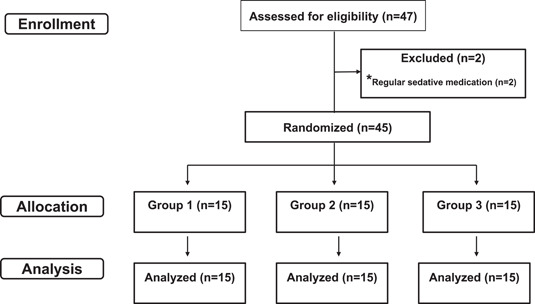
Flow chart of enrollment, allocation, and data analysis

A flow chart of enrollment, allocation, and data analysis is shown in Figure 4.

### 3.1 Demographic and surgical data

Table 1 shows the patient characteristics and the surgical and anesthetic data. There were no significant differences in any of these parameters among the three groups.

**Table 1:** Patient characteristics and surgical and anesthetic data

Basic data	Group 1	Group 2		Group 3	
	(*n* = 15)	(*n* = 15)	*p *Value^a^	(*n* = 15)	*p *Value^a^
Sex, *n* (%)					
Men	7 (46.7)	5 (33.3)	.22	3 (20.0)	.20
Women	8 (53.3)	10 (66.7)		12 (80.0)	
Age, years	36 (11)	29 (9)	.21	31 (13)	.31
Weight, kg	59 (13)	54 (8)	.31	54 (10)	.35
Surgical procedure, *n*					
	Extraction of impacted tooth, 7	Extraction of impacted tooth, 11	.41	Extraction of impacted tooth, 11	.41
	Cyst enucleation, 3	Orthotic device placement, 2		Others, 4	
	Implant placement, 2	Others, 2			
	Others, 3				
Surgery time, min	54 (30)	58 (24)	.41	55 (15)	.72
Local anesthesia, *n* (%)					
Presence	12 (80)	11 (73)	.66	13 (87)	.62
Absence	3 (20)	4 (27)		2 (13)	

*Note*: Values presented as mean (SD), *n*, or *n* (%).

^a^ Versus group 1.

### 3.2 TCI data

The results related to TCI are shown in Table 2. The calculated brain (effect site) concentration when moderate sedation was achieved during induction (value A) (mean ± standard deviation) was smaller in groups 3 and 2 (1.1 ± 0.6 and 1.2± 0.2 μg/ml, respectively) than in group 1 (1.3 ± 0.4 μg/ml); however, this difference was not significant.

**Table 2:** Characteristics related to the TCI, circulatory, and respiratory variables

Parameter data	Group 1	Group 2		Group 3	
	(*n* = 15)	(*n* = 15)	*p *Value^a^	(*n* = 15)	*p *Value^a^
Amnesia during surgery, *n* (%)					
Presence	13 (87)	14 (93)	.72	13 (87)	1.0
Absence	2 (13)	1 (7)		2 (13)	
Value A, μg/ml	1.3 (0.4)	1.2 (0.2)	.52	1.1 (0.6)	.41
Induction time, s	245 (191)	135 (33)*	.006	124 (128)*	.004
Maximum discrepancy, μg/ml	0.5 (0.3)	0.8 (0.2)*	.008	1.0 (1.3)*	.005
Maximum decrease in SBP, %	15 (7)	19 (5)	.24	23 (24)	.14
Maximum decrease in MBP, %	10(6)	14(6)	.29	16(12)	.21
Maximum decrease in DBP, %	7 (5)	9 (7)	.55	11 (7)	.41
Maximum decrease in heart rate, %	5 (7)	9 (10)	.32	10 (8)	.20
Maximum decrease in SpO_2_, %	3.0 (1.0)	3.5 (2.2)	.19	4.9 (6.4)	.21

*Note*: Maximum discrepancy = absolute value of the difference between value B and value A (|value B – value A|). Value A = calculated brain concentration when moderate sedation was recognized during induction, value B = of the regulated TBC values during the operation, the value farthest from value A. Values presented as mean (SD) or *n* (%).

Abbreviations: TCI, targeted controlled infusion; TBC, targeted blood concentration; SBP, systolic blood pressure; MBP, mean blood pressure; DBP, diastolic blood pressure; SpO_2_, arterial oxygen saturation measured by pulse oxymeter.

^a^ Versus group 1.

* *p* < .01.

The induction times (mean ± standard deviation) were significantly shorter in groups 3 and 2 (124 ± 128 s [*p* = .004] and 135 ± 33 s [*p* = .006], respectively) than in group 1 (245 ± 191 s).

The maximum discrepancy (mean ± standard deviation) was significantly larger in group 3 (1.0 ± 1.3 μg/ml) than in group 1 (0.5 ± 0.3 μg/ml) (*p* = .005). The maximum discrepancy was significantly larger in group 2 (0.8 ± 0.2 μg/ml) than in group 1 (0.5 ± 0.3 μg/ml) (*p* = .008).

### 3.3 Cardiovascular and respiratory stability

There were no significant differences in the maximum rate of decrease in blood pressure, heart rate, and SpO_2_ among the three groups (Table 2). No adverse effects such as airway obstruction, apnea, and aspiration were noted in any patients.

## 4 DISCUSSION

In this study, the induction time was shortened due to the elevation of the initial TBC. However, the maximum discrepancy became larger. Both the mean maximum discrepancy and SD for the initial TBC set at 3.0 μg/ml in group 3 (1.0 ± 1.3 μg/ml) seemed to be large enough to impair the validity of this method. TCI using this value cannot enable the prediction of the optimal intraoperative propofol TBC for each individual with a minimal discrepancy during induction. Meanwhile, the maximum discrepancy (0.8 μg/ml) was large, but the SD (0.2 μg/ml) was small in group 2, with an initial TBC set at 2.6 μg/ml. Furthermore, the induction time in group 2 was 45% of that in group 1. Therefore, in cases where we aim to predict the intraoperative TBC according to this maximum discrepancy (0.8 μg/ml), this method with an initial TBC set at 2.6 μg/ml may be acceptable for clinical use to achieve moderate sedation during dental treatment in fearful patients.

Vuyk et al. (1995) evaluated the performance of five computer‐controlled infusion devices in which the pharmacokinetic parameter sets were publicly available, and reported that the measured propofol concentrations exceeded the predicted concentrations in all of them; this difference increased with increasing target propofol concentration. Vuyk et al. (1995) assumed that this was due to the decrease in drug clearance secondary to circulatory suppression or a decrease in the hepatic blood flow. The basic principle behind this method (i.e., the one involving conventional two‐ and three‐compartment mammillary models) assumes that the drug added to the central compartment is completely mixed with the plasma instantaneously and homogeneously and that this mixed plasma appears in the arterial circulation (Morimoto & Harada, 2012; Struys et al., 2007). However, intravenous propofol actually reaches the brain at a high concentration and spreads inhomogeneously (Morimoto & Harada, 2012). Due to this heterogeneity, the sedation obtained by reaching a high concentration of propofol in the brain is likely to be mistaken for the sedation obtained by uniformly diluted propofol. Therefore, in this study, there was a tendency to underestimate the concentration of propofol in the brain at the obtained optimal sedation. Moreover, it is assumed that the higher the initial TBC, the greater this tendency (Vuyk et al., 1995).

The maximum discrepancy of TBC in group 1 (0.5 ± 0.3 µg/ml) is larger than that observed in our previous studies (0.2 ± 0.1 µg/ml in regulation by clinical signs of the sedation level for patients (Fujisawa et al., 2004) and 0.24 ± 0.03 µg/ml in regulation by the BIS values for volunteers (Takuma et al., 2005). The reason for this result may be that we investigated the maximum discrepancy for patients with heterogeneous surgical invasion using objective BIS values.

There were no significant differences in the maximum rate of decrease in circulatory and respiratory parameters among the three groups. Therefore, IVS management seemed to have been performed safely in all groups.

The present study has some limitations. First, as the patients of this study were not limited to those with dental phobia, it remains arguable whether the results could be applied to patients with dental phobia. Second, the results of the present study could hardly be generalizable, because this study was conducted in one oral surgery institution. Third, because Marsh's pharmacokinetics model preloaded in the TCI pump does not include age correction, this method cannot be applied to elderly patients, even if the initial TBC is set at 2.2 µg/ml, which was the minimal setting value in the present study.

In summary, in cases where we aim to predict the intraoperative TBC according to a maximum discrepancy of 0.8 μg/ml, this method with an initial TBC set at 2.6 μg/ml may be acceptable for clinical use, especially to achieve moderate sedation.

## AUTHOR CONTRIBUTIONS

**Toshiaki Fujisawa**: Conceptualization; methodology; project administration; data curation; investigation; writing‐original draft; writing‐review and editing. **Yukie Nitta**: Conceptualization; methodology; data curation; investigation; writing‐review and editing. **Shigeru Takuma**: Conceptualization; methodology; writing‐review and editing.

## CONFLICT OF INTEREST

The authors declare no conflict of interest.

## ETHICS STATEMENT

This study was approved by the local ethics committee (Institutional Review Board of Hokkaido University Hospital, Clinical Study Code: 012‐0298).

## Data Availability

Research data are not shared.
